# Duodenal ampulla neuroendocrine tumor and gastrointestinal stromal tumors in a case of neurofibromatosis type 1: a case report

**DOI:** 10.3389/fonc.2024.1336539

**Published:** 2024-09-30

**Authors:** Tingting Zhang, Nanmu Yang, Peng Zheng, Jiaqi Chen, Bo Meng, Yi Wang, Dapeng Qiu, Xianzhou Zhang, Feng Han, Hao Zhuang, Lu Zheng

**Affiliations:** ^1^ Department of Hepatobiliopancreatic Surgery, The Affiliated Cancer Hospital of Zhengzhou University and Henan Cancer Hospital, Zhengzhou, China; ^2^ Department of Pathology, The Affiliated Cancer Hospital of Zhengzhou University and Henan Cancer Hospital, Zhengzhou, China; ^3^ Department of Medical Oncology, The Affiliated Cancer Hospital of Zhengzhou University and Henan Cancer Hospital, Zhengzhou, China

**Keywords:** case report, neurofibromatosis type 1, gastrointestinal stromal tumors, neuroendocrine tumors, surufatinib

## Abstract

Neurofibromatosis type 1 (NF-1) is commonly associated with a variety of rare tumors. However, no case of multiple gastric gastrointestinal stromal tumors (GISTs) or duodenal ampulla neuroendocrine tumors (NETs) with multiple liver metastases in a patient with NF-1 has yet been reported. Here, we describe a case of a 55-year-old female patient with NF-1 whose serum Pro-Gastrin-Releasing Peptide (pro-GRP) levels were elevated. Gastrointestinal endoscopy and biopsy showed duodenal papilla space-occupying mass, and the pathological diagnosis turned out to be neuroendocrine tumors (NETs). During surgical exploration, multiple tumors were found on the serosal surface of the stomach and numerous miliary metastases in the liver. Following histopathological examination, it was determined that the liver metastases were NF-1 and the tumors in the gastric wall were GISTs. The patient benefited from targeted therapy and had an uneventful hospital stay. In this case, we emphasize treating patients with neurofibromatosis type 1 who exhibit abdominal symptoms with a high degree of clinical suspicion and performing thorough evaluations to rule out multiple tumors.

## Introduction

Neurofibromatosis type 1 (NF-1) is an autosomal dominant neurocutaneous disorder with a prevalence of 1 per 4,000 individuals, which arises as a result of *NF1* gene mutation, located on chromosome 17q11.2 (1). NF-1 increases the risk of malignancy and decreases life expectancy, in addition to being associated with a broad range of clinical presentations. Café-au-lait spots and peripheral neurofibromas are commonly observed (2). Previous studies have reported that *NF1* gene mutation can lead to Ras pathway abnormalities, which may also result in peripheral neurilemmomas, central nervous system tumors, stromal tumors, neuroendocrine tumors, and other benign and malignant tumors.

Gastrointestinal stromal tumors (GISTs) are intestinal stromal tumors originating from the interstitial cells of Cajal, which are found in the intestine. GISTs are relatively common, with prevalences estimated to vary from 5% to 30% in patients with NF-1, usually affecting elderly individuals with a median age of approximately 60–65 years (3). GISTs can develop anywhere in the gastrointestinal tract. The stomach accounts for approximately 60% of all cases, while the duodenum and rectum account for only approximately 5% (4). However, in the case of NF-1, approximately 90% of GISTs are found in the small intestine, while only 5.4% are located in the stomach (5).

NETs arise from the endocrine cells of the gastrointestinal tract, which are enterochromaffin cells derived from the crypts of Lieberkühn in the mucosa and submucosa (6). NETs typically originate in the gastroenteropancreatic system. Duodenal ampulla NETs are extremely uncommon, with an incidence of <1% (7).

We report an extremely rare case of multiple gastric GISTs and duodenal ampulla NETs, along with multiple liver metastases, in a patient with NF-1. While instances of these tumor types have been documented in individuals with NF-1, the coexistence of both NETs on the duodenal ampulla and GISTs in the stomach is exceedingly uncommon.

## Case report

A 55-year-old female patient with NF-1 presented to our hospital due to a suspected space-occupying lesion in the ampulla. The presence of café-au-lait spots on the body and limbs, multiple neurofibromas over the trunk and face, her *NF1* gene mutation, and family history of NF-1 in a first-degree relative (sister) confirmed the diagnosis of NF-1. The patient denied nausea, emesis, chest pain, shortness of breath, syncope, or other symptoms at the time. Physical examination revealed generalized icterus, but no palpable abdominal lump was detected and Murphy’s sign was negative.

Initial laboratory analysis of this patient showed high levels of total bilirubin (TBIL), 28.4 µmol/L; direct bilirubin (DBIL),19.1 µmol/L; ALT, 226 µmol/L; AST, 229 µmol/L; ALP, 610 µmol/L; GGT, 933 µmol/L; blood ammonia, 76.5 µmol/L; and pro-GRP, 334 pg/mL.

**Figure 1:**
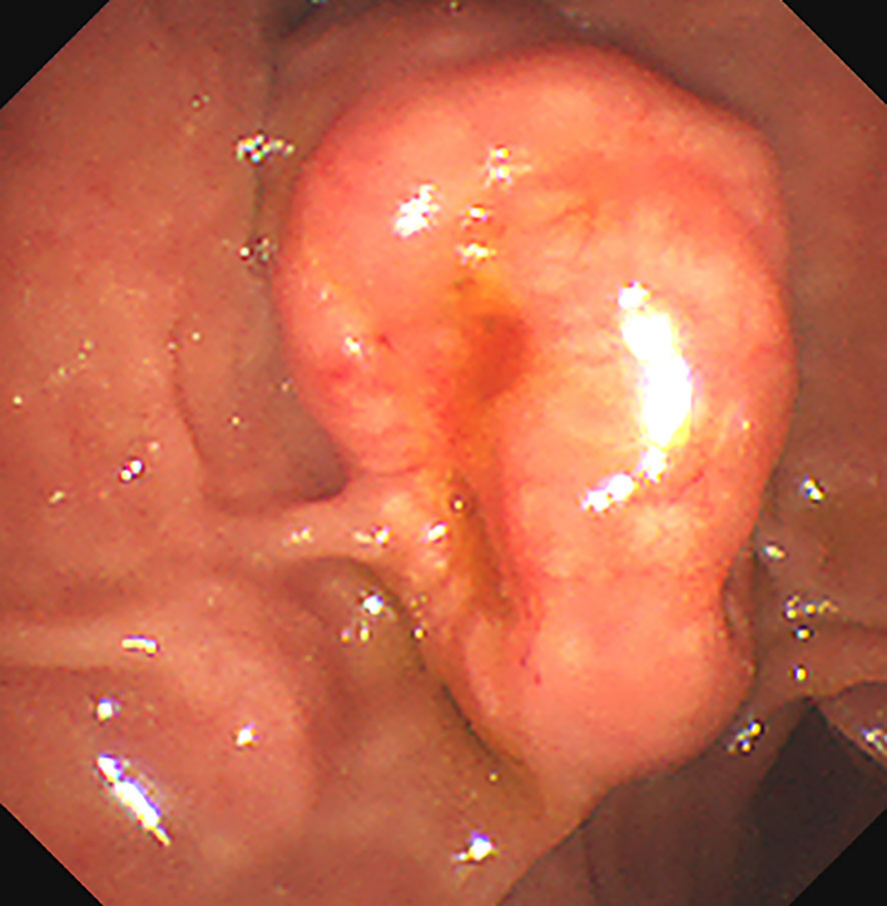
The Esophagogastroduodenoscopy (OGD) shows a bulging mass at the duodenal papilla region. There are depression and erosion at the opening of the protruding duodenal papilla.

**Figure 2:**
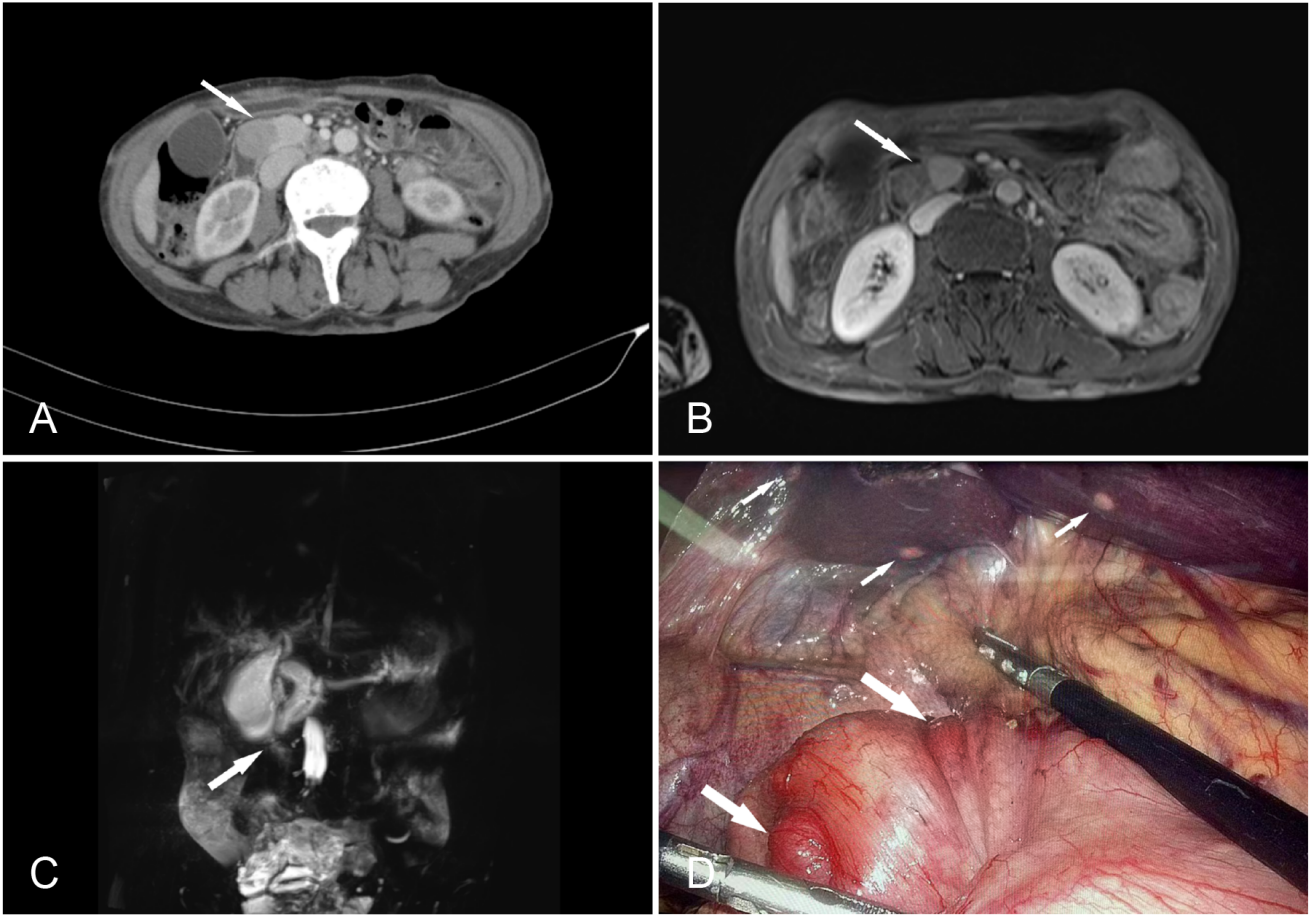
**(A)** Contrast-enhanced computed tomography of the abdomen showing a prominent enhancing soft tissue mass (arrow), suggesting a diagnosis of a duodenal ampulla tumor. **(B)** MRI venous time shows a mass at the pancreas head and ampullae area (arrow), approximately 38 mm × 20 mm × 28 mm. **(C)** Magnetic resonance cholangiopancreatography (MRCP) showing a significant dilation of the intra- and extrahepatic bile ducts and the main pancreatic duct, with visible truncation at the junction of the two (arrow). **(D)** During the operation, multiple small nodules were identified on the serosal surface of the stomach (large arrows) and multiple metastasis on the liver (small arrows).

Gastrointestinal endoscopy and biopsy revealed the presence of a duodenal papilla that occupied space and exhibited depression and erosion at the raised opening. Pathological diagnosis confirmed the presence of NETs (G1) (**Figure 1**). Computed tomography (CT) of the abdomen with contrast revealed several lesions in the duodenal ampulla area, along with dilation of both intrahepatic and extrahepatic bile ducts and pancreatic ducts (**Figure 2A**). Additionally, numerous calcifications were detected in the liver. The diagnosis of NF-1 was further confirmed by the presence of soft tissue nodules and mass shadows on the skin and subcutaneous areas of the head, neck, and chest. The results of nuclear magnetic resonance imaging (MRI) indicated an abnormal signal in the pancreatic head region, which suggested a diagnosis of NETs (**Figure 2B**). Magnetic resonance cholangiopancreatography (MRCP) revealed marked dilation of both the intra- and extrahepatic bile ducts and the main pancreatic duct. Visible truncation was observed at the intersection of the two ducts (**Figure 2C**).

**Figure 3:**
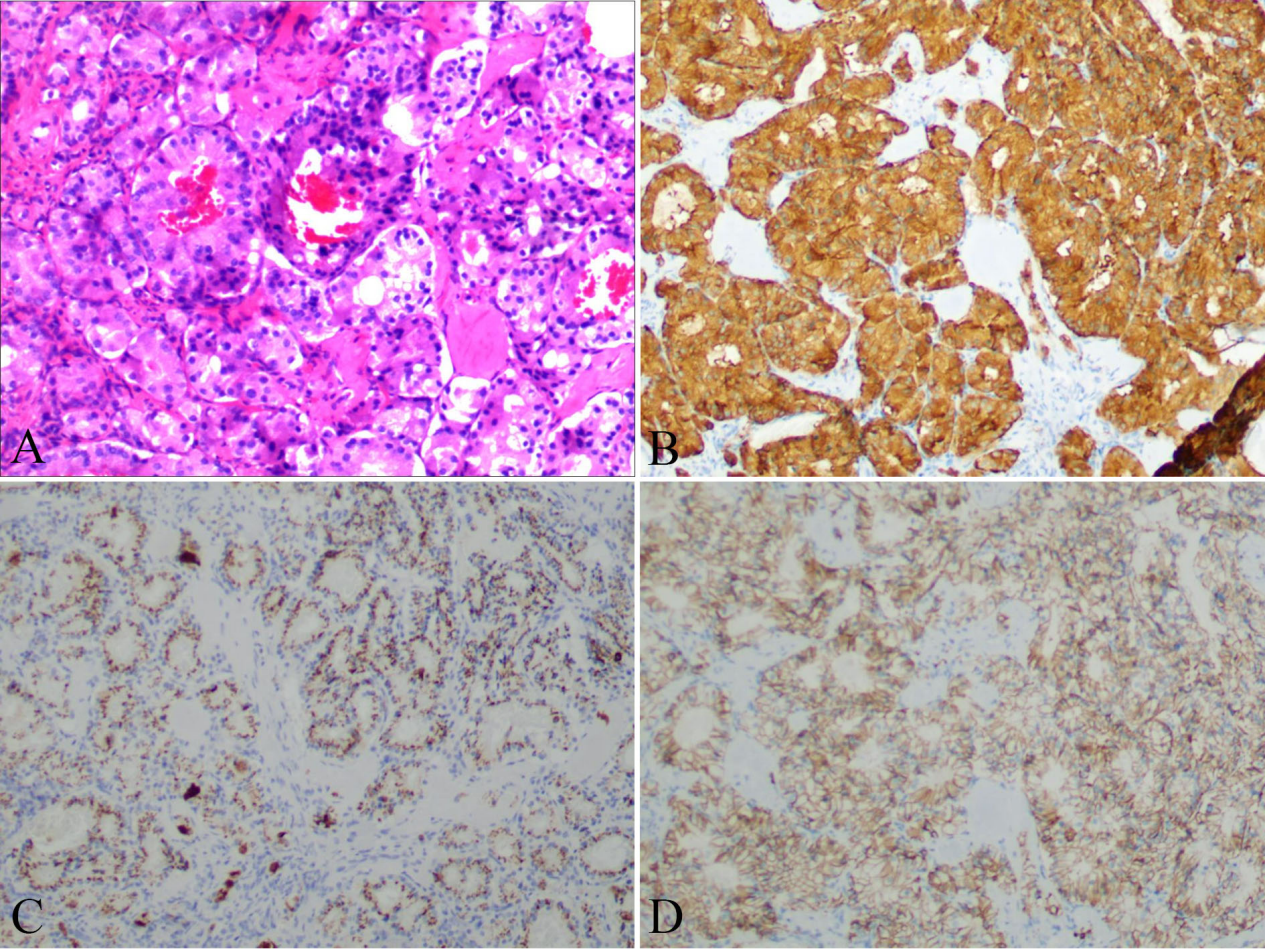
Liver nodules distribute an adenoid structure **(A)** with positive expression of SyN **(B)**, CgA **(C)**, and CD56 **(D)**.

**Figure 4:**
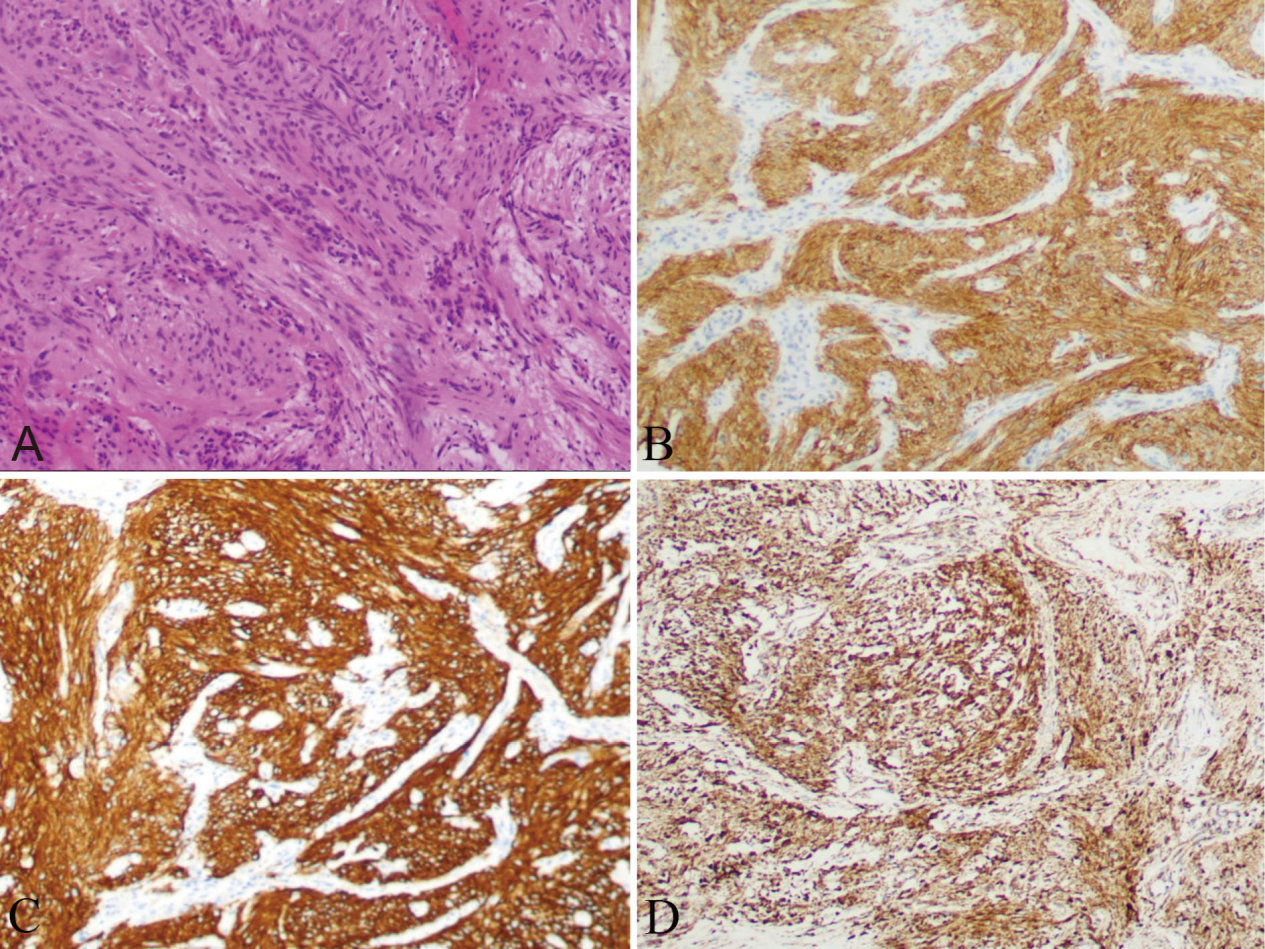
Nodules on the serosal surface of stomach showing a trabecular pattern, suggesting GIST **(A)**, with a positive expression of CD117 **(B)**, DOG-1 **(C)**, and SDHB **(D)**.

The patient was clinically diagnosed with GISTs and NETs in NF-1. The patient underwent laparoscopic pancreatoduodenectomy after providing written informed consent. During the exploratory operation, we discovered several tumors on the serosal surface of the stomach and multiple miliary metastases (0.5–0.8 cm) in the liver (**Figure 2D**). Two liver lesions and one gastric wall tumor were surgically excised to obtain a pathological diagnosis. Histopathological examinations revealed that the liver metastases were composed of NETs with a Ki67 value of 1%, indicating a low grade (**Figure 3**). The tumors observed in the gastric wall were identified as GISTs (**Figure 4**). Surgical resection could not be performed due to the presence of multiple developed metastatic tumors. The patient was transferred to the internal medicine department for treatment. Blood tests showed high levels of vascular endothelial growth factor (VEGF) (215.0 pg/mL) and pro-GRP (334.9 pg/mL). The patient underwent targeted therapy with 300 mg of surufatinib daily and symptomatic treatment based on the results of the examination and the patient’s condition.

## Discussion

GISTs are predominant mesenchymal tumors observed in individuals with NF-1. Individuals with NF-1 are more likely to develop GISTs (7%) than the general population. It mostly occurs in the small intestine (8). However, our case involved GISTs localized to the stomach with multiple lesions. Ampullary NETs are reported to be exceedingly rare, with an incidence <0.05% among GI NETs (9). Approximately 26% of NETs are associated with neurofibromatosis (10). Although both NETs and GISTs are uncommon tumors, their coexistence is even rarer, with only two reported cases (11, 12). These clinical entities should be considered when patients are diagnosed with NF-1.

Ampullary NETs have a higher tendency to exhibit high-grade tumors and lymph node metastases (7). Jaundice (65%) and pain (31%) are the most frequently reported symptoms (13). Our finding of gastrinoma contrasts with the most prevalent type of peri-ampullary NETs observed in patients with NF-1, which is somatostatinoma (40%). Compared to duodenal NETs, ampullary NETs are associated with poorer overall survival rates. Pancreaticoduodenectomy is the recommended therapeutic approach for well-differentiated ampullary carcinoid tumors measuring more than 2 cm and for ampullary neuroendocrine carcinomas (14). GISTs in NF-1 usually involve multiple occurrences in the small bowel rather than sporadic GISTs (15). Spindle cell morphology is observed in the majority of tumors, along with a lower mitotic rate and Ki67 index, as per histological examination (16). Due to their resistance to imatinib mesylate therapy caused by the absence of c-kit mutations, surgical intervention remains the sole curative treatment option (17). In this study, we presented a unique case of gastric GISTs associated with NF-1. The patient underwent imatinib mesylate therapy followed by surgical intervention.

Currently, there are no screening guidelines for gastrointestinal tumors in patients with NF-1, and surgery is the preferred treatment for GISTs and NETs in NF-1. However, if surgical treatment is not administered, the treatment options become significantly limited. Gastrointestinal manifestations in individuals with NF-1 typically occur in middle-aged and older individuals. GISTs and NETs in NF-1 are usually asymptomatic in the initial stages, often diagnosed at an advanced cancer stage; thus, patients miss the chance for surgical intervention. In the present work, the patient exhibited multiple tumors, including GISTs and NETs. She was initially asymptomatic and therefore missed the optimal treatment window, leading to an unfavorable prognosis. Considering the potential concealment and elevated risk of tumor development resulting from genetic abnormalities, we recommend conducting regular examinations on elderly patients even in the absence of adequate clinical evidence supporting this screening practice. Contrast-enhanced computed tomography (CT) and magnetic resonance imaging (MRI), along with the presence of other potential diagnostic tumor biomarkers, are highly sensitive diagnostic modalities. Despite the general rarity of these tumors, it is crucial to maintain a high level of suspicion and ensure early diagnosis in asymptomatic patients with NF-1.

Furthermore, our case highlights one challenge inherent in the diagnosis of gastrointestinal tumors associated with NF-1. Proper diagnosis of abdominal manifestations of NF-1 is essential to finding appropriate treatment and preventing severe organic complications associated with tumor masses. The definitive diagnosis of GISTs or NETs is typically confirmed through immunohistochemistry following a tissue biopsy. Due to the potential development of various rare tumors in NF-1, obtaining biopsies from multiple locations is recommended for definitive histopathological diagnosis.

## Conclusions

In this study, it was found that during the early stages, GISTs and NETs in patients with NF-1 often remain asymptomatic but tended to have already metastasized by the time patients sought treatment. Despite the lack of current screening guidelines for gastrointestinal tumors in patients with NF-1, early diagnosis and management play a crucial role in reducing both mortality and morbidity. While the definitive diagnosis of GISTs or NETs is typically confirmed through immunohistochemistry after a tissue biopsy, it is advisable to obtain biopsies from multiple locations to achieve a conclusive histopathological diagnosis.

## Data Availability

The original contributions presented in the study are included in the article/supplementary material. Further inquiries can be directed to the corresponding authors.
